# Multidrug-Resistant *Acinetobacter baumannii*: Resistance Mechanisms, Emerging Therapies, and Prevention—A Narrative Review

**DOI:** 10.3390/antibiotics15010002

**Published:** 2025-12-19

**Authors:** Ioana Adelina Stoian, Bianca Balas Maftei, Carmen-Elena Florea, Alexandra Rotaru, Constantin Aleodor Costin, Maria Antoanela Pasare, Radu Crisan Dabija, Carmen Manciuc

**Affiliations:** 1 Grigore T. Popa University of Medicine and Pharmacy, 700115 Iasi, Romania; 2 Department of Morphofunctional Sciences I, Grigore T. Popa University of Medicine and Pharmacy, 700115 Iasi, Romania; 3 Clinical Hospital of Pneumology, 700115 Iasi, Romania; 4 “Sfânta Parascheva” Clinical Hospital for Infectious Diseases, 700116 Iasi, Romania

**Keywords:** *Acinetobacter baumannii*, carbapenem resistance, multidrug resistance, extensively drug-resistant, OXA carbapenemases, cefiderocol, sulbactam–durlobactam, ICU infections, antimicrobial therapy

## Abstract

*Acinetobacter baumannii* is a leading intensive care unit (ICU) pathogen associated with high rates of carbapenem resistance and poor clinical outcomes. This narrative review synthesizes recent clinical, microbiological, and pharmacokinetic/pharmacodynamic (PK/PD) evidence regarding resistance mechanisms and therapeutic strategies. A literature review was performed in PubMed, Scopus, and Web of Science (January 2015–August 2025), focusing on multidrug-resistant (MDR) and extensively drug-resistant (XDR) strains, ICU-acquired infections, and pivotal trials involving cefiderocol and sulbactam–durlobactam. Resistance is driven by OXA-type carbapenemases (notably OXA-23/24/58), efflux systems (AdeABC/IJK/FGH), porin alterations (CarO, Omp33–36), and lipopolysaccharide (LPS) modifications conferring colistin resistance. Management options include polymyxins, optimized tigecycline dosing, β-lactam/β-lactamase inhibitors, and newer agents such as cefiderocol and sulbactam–durlobactam, though mortality and safety outcomes vary across trials. A comparative table is included, summarizing antimicrobial mechanism coverage, PK/PD parameters, and adverse effects to support regimen selection in ventilator-associated pneumonia (VAP) and bacteremia. Optimized, multimodal approaches integrating timely diagnostics, targeted combination therapies, infection prevention, and antimicrobial stewardship are essential to improve outcomes and limit the spread of MDR and XDR *A. baumannii*.

## 1. Introduction

*Acinetobacter baumannii* (*A. baumannii*) is a Gram-negative, aerobic, nonmotile coccobacillus commonly found in the human and animal microbiota, as well as in environmental sources such as water, soil, sewage and hospital environments [1,2]. Due to its remarkable ability to develop antimicrobial resistance and persist in clinical settings, *A. baumannii* has been classified among the ESKAPE pathogens—a group of six highly virulent and drug-resistant organisms that collectively represent the leading cause of hospital-acquired infections worldwide. The acronym ESKAPE stands for *Enterococcus faecium*, *Staphylococcus aureus*, *Klebsiella pneumoniae*, *Acinetobacter baumannii*, *Pseudomonas aeruginosa*, and *Enterobacter* species, all known for their capacity to “escape” the effects of most conventional antibiotics and to thrive in high-risk healthcare environments [3]. In recent decades, this opportunistic pathogen has increasingly been linked with healthcare-associated infections, particularly in intensive care units (ICUs) and among immunocompromised patients. *A. baumannii* can lead to a broad spectrum of serious infections, ranging from respiratory and bloodstream infections to meningitis and urinary tract involvement, especially in hospitalized patients. The primary mode of transmission is via healthcare workers’ hands, though aerosol and droplet spread have also been documented [1,2].

According to the World Health Organization’s 2024 update, carbapenem-resistant *Acinetobacter baumannii* (CRAB) remains classified as a “critical priority” pathogen due to its high mortality and resistance to nearly all available antibiotics [4]. Globally, CRAB accounts for approximately 80% of hospital-acquired and ventilator-associated pneumonia (HAP/VAP) cases, with prevalence exceeding 90% in Southeast Asia, compared to less than 25% in North America and Western Europe. In 2023, the EU reported a CRAB bloodstream infection incidence of 2.98 per 100,000 population, with carbapenem resistance rates ranging from under 1% in Northern Europe to over 50% in countries such as Romania, Greece, and Italy [5].

Similar resistance dynamics have also been reported in *Enterobacterales* isolates from the same region, especially in urinary tract infections. Recent findings documented the emergence of multidrug resistance and colistin resistance, including during the COVID-19 pandemic [6,7].

A brief overview of the taxonomic classification and evolutionary background of *A. baumannii* is provided below to support the understanding of its clinical relevance and global spread.

The formal taxonomy of *A. baumannii* was established in 1986 by Bouvet and Grimont through DNA–DNA hybridization techniques, which differentiated it from related species within the *Acinetobacter calcoaceticus–baumannii* (*ACB*) complex. Since the 1970s, *A. baumannii* has been recognized as a significant healthcare-associated pathogen, notable for its ability to survive harsh environmental conditions, including desiccation, and its increasing antimicrobial resistance. These characteristics contribute to its ongoing clinical importance [8,9].

Many reviews focus either on microbiological mechanisms or treatment guidelines in isolation. However, few integrate these aspects with emerging therapeutic agents and infection control strategies, despite the complex interplay between resistance mechanisms, clinical decision-making, and outcomes. This narrative review addresses this gap by providing an integrated, clinically oriented synthesis of resistance mechanisms, emerging antimicrobial therapies, and infection control strategies for multidrug-resistant *A. baumannii*. With a focus on severe ICU-acquired infections such as ventilator-associated pneumonia and bloodstream infections, it aims to support therapeutic decision-making by summarizing drug activity, pharmacological profiles, and clinical efficacy of novel agents.

## 2. Materials and Methods

### 2.1. Search Strategy

This work is a structured narrative review intended to synthesize key microbiological, clinical, and therapeutic aspects of multidrug-resistant (MDR) and extensively drug-resistant (XDR) *Acinetobacter baumannii*, with emphasis on ICU-acquired infections. Although a targeted and reproducible literature search was conducted, this review does not follow PRISMA methodology and should not be considered a systematic review. The database search was selective rather than exhaustive, guided by clinical relevance and expert judgment. A PRISMA-style flow diagram is included solely as a descriptive tool to illustrate the identification, screening, and selection of articles, without implying adherence to the full PRISMA framework. The search covered the period from January 2015 to August 2025 to ensure that the evidence reflected current clinical practice, particularly with respect to emerging therapeutic agents and resistance mechanisms in ICU-acquired infections. Earlier studies, while historically important, were not systematically included in order to maintain a consistent and contemporary, practice-oriented evidence base.

In PubMed, the following Boolean string was used as a representative search example: (“*Acinetobacter baumannii*” OR “CRAB” OR “multidrug-resistant *Acinetobacter*”) AND (“carbapenem resistance” OR “OXA carbapenemase” OR “cefiderocol” OR “sulbactam–durlobactam”) AND (“ICU” OR “ventilator-associated pneumonia” OR “bacteremia” OR “antimicrobial therapy”). Medical Subject Headings (MeSH) were consulted but not applied systematically, as the narrative nature of this review prioritized flexibility and inclusion of recent terms not yet indexed. Reference lists of key studies and reviews were manually screened for additional relevant articles. Duplicate records were removed manually during the selection process. Gray literature, including preprints, non-peer-reviewed reports, and conference abstracts, was excluded to ensure the reliability of included sources. The data analysis was performed using Microsoft Excel and PowerPoint 2016.

### 2.2. Inclusion and Exclusion Criteria

Studies were included if they focused on infections caused by multidrug-resistant (MDR), extensively drug-resistant (XDR), or carbapenem-resistant (CRAB) *Acinetobacter baumannii* strains, particularly in the context of healthcare-associated or ICU-acquired infections. Eligible articles had to report clinical, microbiological, or therapeutic data related to *A. baumannii* and its antimicrobial management. Priority was given to human clinical studies (observational or interventional studies, systematic reviews, meta-analyses, and international treatment guidelines). Selected in vitro and pharmacodynamic/pharmacokinetic studies were also included when they provided essential information on antimicrobial mechanisms of action or drug pharmacological profiles relevant to MDR/XDR/CRAB *A. baumannii*.

Exclusion criteria included:

- Non-peer-reviewed sources (e.g., editorials, narrative reviews without original data, opinion papers)
- Conference abstracts lacking full data or without an associated full-text article
- Non-English articles (unless a verified translation was available)
- Studies conducted exclusively in vitro that did not provide clinically relevant information on resistance mechanisms or drug properties
- Articles not specifically addressing MDR/XDR/CRAB *A. baumannii* or unrelated to antimicrobial resistance and ICU/healthcare-associated infections

### 2.3. Data Extraction and Synthesis

Relevant data were independently extracted by four authors and included information on antimicrobial resistance mechanisms, clinical outcomes, therapeutic options, and pharmacological properties of emerging agents. Any discrepancies or differences in interpretation were resolved through discussion and mutual agreement. 

**Figure 1:**
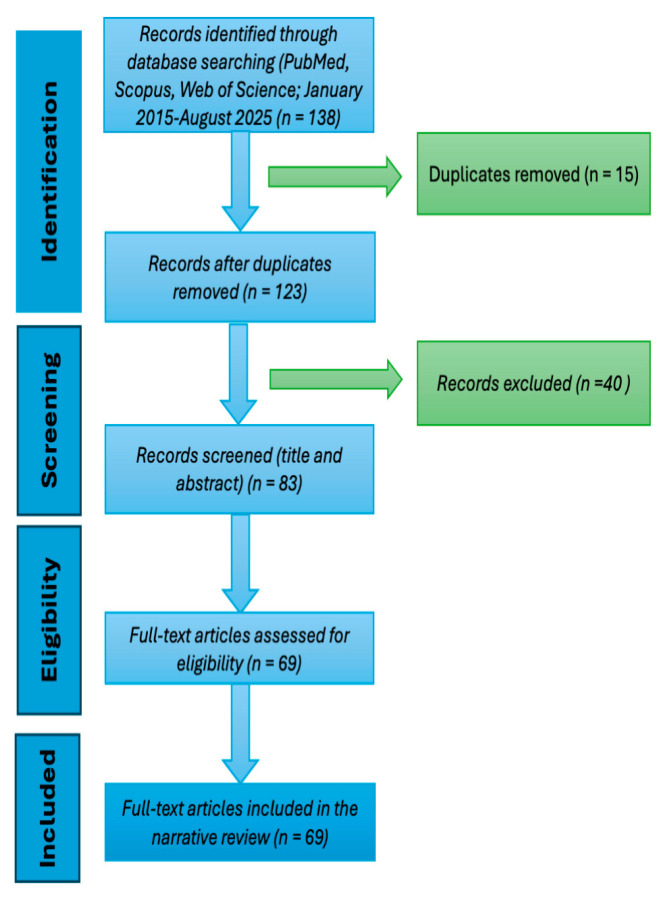
Flow diagram illustrating the literature search and study selection process.

Given the narrative nature of this review, study heterogeneity was not assessed statistically but was addressed through thematic organization of content. Synthesized data were structured primarily by clinical context (e.g., ICU infections, pneumonia, bacteremia) and resistance mechanism (e.g., OXA enzymes, efflux pumps) to ensure a coherent and clinically relevant presentation (Figure 1).

## 3. Epidemiology and Clinical Significance

### 3.1. Community-Acquired Infections Versus Healthcare-Associated Infections

Although mainly a healthcare-associated pathogen, *A. baumannii* is increasingly reported as a cause of community-acquired pneumonia (CAP), especially in tropical and subtropical regions [1]. Reports from Northern Australia, Taiwan, and Brazil link its emergence to environmental factors like temperature and humidity. Common risk factors include alcoholism, diabetes, and chronic lung disease [10]. These infections often follow a fulminant course. A study from Northern Australia reported mortality rates exceeding 60% in patients with community-acquired *A. baumannii* pneumonia, many of whom presented with septic shock and required early mechanical ventilation [11].

While community-acquired *A. baumannii* infections are less common, they highlight the organism’s clinical versatility and underscore the importance of understanding its full pathogenic spectrum before focusing on the more prevalent healthcare-associated strains. Its ability to persist on dry surfaces and rapidly acquire antimicrobial resistance mechanisms has elevated it from an opportunistic colonizer to a global healthcare priority [1,12].

Global surveillance shows that CRAB represents 30–70% of hospital isolates, with the highest rates in Southeast Asia, Eastern Europe, and Latin America [13]. The pathogen’s inclusion in the ESKAPE group underscores its role in driving antimicrobial resistance worldwide [14].

### 3.2. Global Resistance Trends

The WHO classifies CRAB as a critical-priority pathogen, with the 2024 WHO list reaffirming its status among the top threats due to limited therapeutic options [15]. In high-burden regions, MDR/XDR *A. baumannii* strains account for over 70% of isolates [16,17,18,19,20]. However, this figure varies widely depending on local epidemiology, with countries such as Finland and Norway consistently reporting resistance rates below 10%. In critically ill patients, especially those with VAP or bloodstream infections, mortality ranges from 40% to 80% [16,17,18,19,20].

Longitudinal surveillance data show a clear rise in CRAB prevalence over time. Historical data from the early 2000s indicate that carbapenem resistance among *A. baumannii* isolates was substantially lower in many regions, with some studies reporting rates in the range of 30–36% in Europe between 2013 and 2017 [21]. In contrast, more recent meta-analyses encompassing data from 2020 to 2023 reveal that overall carbapenem resistance among *A. baumannii* isolates has risen to approximately 74–81% globally, with highest resistance rates observed in Asia, Africa, and parts of southern Europe [22]. Certain regional studies further report local resistance rates reaching ~90% in the Middle East and Mediterranean area, underscoring the increasing burden in these settings [23]. These longitudinal data demonstrate a clear upward trend in CRAB prevalence over the past decade, reflecting the growing challenge of carbapenem resistance in clinical settings and the need for enhanced antimicrobial stewardship and infection control strategies.

Moreover, a 2021 European Centre for Disease Prevention and Control (ECDC) report indicated that more than 15% of intensive care patients who stay over 48 h acquire at least one healthcare-associated infection, underscoring the ongoing relevance of *A. baumannii* as a nosocomial pathogen [24]. Seasonal variation also plays a role, with higher incidence during warmer months—suggesting that climate factors such as temperature and humidity may influence pathogen persistence and transmission [2]. At the regional level, resistance rates show remarkable heterogeneity, with some of the highest values recorded in Eastern Europe, particularly in Romania.

### 3.3. Regional Data from Romania

In Romania, surveillance data consistently demonstrate alarming multidrug and carbapenem resistance rates among clinical *A. baumannii* isolates, particularly within intensive care units. The pathogen has become a dominant cause of ventilator-associated and bloodstream infections, reflecting intense antibiotic pressure and clonal persistence within hospital environments (Table 1).

Taken together, these data highlight *A. baumannii* as a resilient and rapidly evolving pathogen that continues to challenge healthcare systems worldwide. Understanding local resistance patterns—such as those described in Romania—is essential to guiding both empirical therapy and infection control policies. The regional variability in resistance underscores the need for tailored surveillance and intervention strategies, especially in high-risk areas such as Romania.

## 4. Mechanisms of Antimicrobial Resistance in *Acinetobacter baumannii*

Misuse and overuse of antibiotics have been central drivers of the escalating resistance observed in *A. baumannii*. In its 2024 update, the World Health Organization expanded the list of bacterial priority pathogens to include 24 agents across 15 bacterial families, reaffirming the critical threat posed by *A. baumannii*, especially carbapenem-resistant strains [15].

Therapeutic combinations, including ampicillin-sulbactam and carbapenems, have shown effectiveness in targeting MDR *A. baumannii* in both lab and clinical contexts. Nonetheless, resistance continues to evolve, necessitating new therapeutic approaches and innovative drug development strategies [29,30,31].

The multidrug-resistant phenotype of *A. baumannii* results from the interplay of several adaptive mechanisms acting simultaneously rather than independently. Understanding how these mechanisms converge is essential to designing effective therapeutic strategies. To better understand the multidrug-resistant phenotype of *A. baumannii*, this review highlights the primary resistance mechanisms: enzymatic degradation, altered permeability, active efflux, and target site modifications.

### 4.1. β-Lactamase Production and Carbapenem Resistance

The main resistance mechanism in *A. baumannii* is the production of β-lactamases. These enzymes inactivate β-lactam antibiotics by hydrolyzing the β-lactam ring. Among them, class D carbapenem-hydrolyzing oxacillinases (OXA-type) are the most clinically relevant. The most widespread are OXA-23, OXA-24/40, OXA-58, and, more rarely, OXA-143 and OXA-235. These enzymes confer high-level resistance to carbapenems and are often encoded by plasmids or transposons, facilitating horizontal gene transfer [32].

In addition to class D enzymes, some *A. baumannii* isolates produce class B metallo-β-lactamases (MBLs), such as IMP, VIM, and NDM, although these are less common. Unlike OXA enzymes, MBLs require zinc as a cofactor and are not inhibited by β-lactamase inhibitors like sulbactam or tazobactam [32].

This diversity of β-lactamases complicates both diagnosis and treatment. Routine laboratory testing may underestimate resistance when gene detection is not included, and treatment failure is common when carbapenems are used empirically [32].

Table 2 summarizes the classification (Ambler scheme), enzymatic mechanisms, inhibition profiles, and relevance to clinical isolates of *A. baumannii*.

The enzymes listed below represent the most frequently encountered carbapenemases in *A. baumannii*, including both intrinsic and acquired types (Table 3).

### 4.2. Efflux Pumps

Efflux pumps actively extrude antibiotics from the bacterial cell, contributing significantly to multidrug resistance [33]. These active transport systems, particularly those of the Resistance-Nodulation-Division (RND) family—*AdeABC*, *AdeIJK*, and *AdeFGH*—play a key role in MDR by extruding multiple antibiotics, including aminoglycosides, fluoroquinolones, β-lactams, and tigecycline [34,35].

Recent functional analyses confirm that gene knockouts of these systems reduce resistance levels, biofilm formation, and virulence expression [35]. The *AdeABC* system, driven by *adeRS* regulatory elements, is most strongly associated with MDR phenotypes. Overexpression of these pumps is closely linked to therapeutic failure, particularly in ventilator-associated pneumonia [35].

Several efflux pump systems—especially from the RND, MFS, and MATE families—contribute significantly to multidrug resistance in *A. baumannii*. Table 4 lists the key systems, their gene components, and the targeted antimicrobials.

### 4.3. Outer Membrane Porins

Porin loss reduces outer membrane permeability and limits antibiotic entry, enhancing resistance. Key porins implicated in *A. baumannii* include OmpA, Omp33–36, CarO, and OprD. Among them, CarO is specifically involved in imipenem uptake, and its inactivation has been strongly associated with carbapenem resistance [36]. Dynamic structural studies of Omp33–36 suggest this porin adjusts its permeability in response to antibiotic stress. Combined with efflux pump overexpression, porin alterations form a dual barrier mechanism—reducing antibiotic entry while enhancing active extrusion [9]. Loss, mutation, or downregulation of specific porins impairs antibiotic influx and contributes to resistance, as shown in Table 5.

### 4.4. Fluoroquinolone Resistance via Target Site Mutations

Resistance to fluoroquinolones is primarily driven by mutations in the quinolone resistance-determining regions (QRDRs) of gyrA and parC [37,38]. The most common substitutions, Ser83Leu (GyrA) and Ser80Leu (ParC), reduce fluoroquinolone binding and are strongly associated with high-level resistance [37,38]. In a 2021 study, over 70% of fluoroquinolone-resistant *A. baumannii* isolates harbored these mutations, often in combination with plasmid-mediated resistance genes such as *qnrA* and *aac(6′)-Ib-cr*, which further amplified resistance levels [37]. More recently, rare but clinically relevant mutations have been identified in gyrB and parE (e.g., *gyrB* Asp426→Asn, *parE* Ile444→Thr), particularly in strains with MIC values exceeding 128 µg/mL for ciprofloxacin [38]. These findings highlight the complex and dynamic nature of fluoroquinolone resistance in *A. baumannii*, driven by both chromosomal mutations and plasmid-mediated mechanisms.

Taken together, these resistance determinants highlight the extraordinary genomic plasticity of *A. baumannii*, which allows rapid adaptation to antibiotic pressure through both chromosomal mutations and horizontal gene transfer.

**Figure 2:**
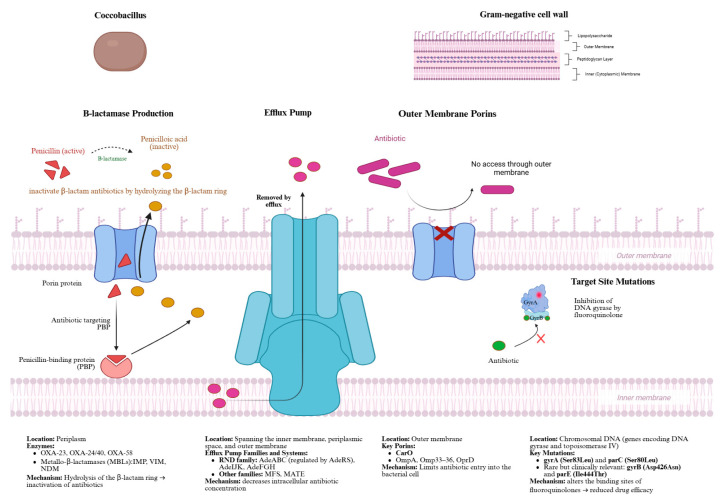
Mechanisms of antibiotic resistance in *Acinetobacter baumannii*. PBP—Penicillin Binding Protein; OXA—Oxacillinase; IMP—Imipenemase RND—Resistance Nodulation Division; VIM—Verona integron-encoded metallo-β-lactamase; NDM—New Delhi metallo-β-lactamase; Ade—Multidrug efflux pump system; MFS—Major Facilitator Superfamily; MATE—Multidrug and Toxic Compound Extrusion; MFS—Major Facilitator Superfamily; CarO—Carbapenem-Associated outer membrane protein O; OmpA—Outer Membrane Protein A; Omp33-36—Outer membrane porin 33–36 kDa; OprD—Outer Membrane Protein D; DNA—deoxyribonucleic acid; Gyr A—DNA gyrase subunit A; GyrB—DNA gyrase subunit B; parC—Topoisomerase IV subunit A gene; parE—Topoisomerase IV subunit B gene; Asp—Aspartic acid; Asn—Asparagine; Ile—Isoleucine; Thr—Threonine; Leu—Leucine; Ser—Serine. Created in BioRender. Stoian, A. (2026) https://BioRender.com/sznrk83. Accessed on 16 December 2025.

To enhance clarity, Figure 2 summarizes the main resistance mechanisms and patterns associated with MDR and XDR *Acinetobacter baumannii*.

### 4.5. Clinical Implications

In the context of ventilator-associated infections (VAP), these resistance mechanisms severely limit the efficacy of empirical therapy, often necessitating last-resort agents and contributing to increased ICU length of stay and mortality. Co-expression of *AdeABC* efflux pumps and OXA-type carbapenemases has been correlated with therapeutic failure to agents such as tigecycline and meropenem, particularly in ventilated patients [32,34,35].

A deeper understanding of these resistance mechanisms is critical for the development of effective therapeutic combinations and novel agents. Future efforts should focus on integrating molecular resistance profiling into clinical decision-making, enabling personalized antimicrobial therapy and timely infection control interventions. This approach aligns with current efforts to personalize antimicrobial therapy based on resistance genotypes.

## 5. Current Treatment Options and Emerging Therapies

Treating *A. baumannii* infections, particularly MDR and XDR strains, is a major clinical challenge due to limited options and poor outcomes. In addition, pharmacokinetic issues, nephrotoxicity, and limited clinical trial data complicate their use in clinical practice. As a result, clinicians are often forced to rely on off-label regimens or combination strategies with variable success. This section provides a structured overview of both established and emerging antimicrobial agents, including siderophore cephalosporins, β-lactamase inhibitors, and last-resort options. Key pharmacological properties, clinical evidence and safety considerations are discussed to support informed therapeutic decision-making in the era of antimicrobial resistance.

### 5.1. Colistin

Colistin remains a widely used last-line agent for the treatment of pneumonia caused by carbapenem-resistant *A. baumannii* (CRAB), particularly in ventilator-associated pneumonia (VAP) among critically ill patients [39,40]. Despite in vitro activity, intravenous colistin often yields poor outcomes due to low lung penetration, nephrotoxicity, and altered pharmacokinetics in ICU patients [41].

Recent observational studies suggest that adjunctive inhaled (nebulized) colistin, when added to systemic therapy, may enhance treatment efficacy in VAP. A 2023 meta-analysis by Zhang et al. concluded that inhaled colistin as adjunctive therapy significantly improved microbiological eradication and oxygenation parameters, with no increase in nephrotoxicity compared to intravenous therapy alone [40]. In a 2024 randomized controlled trial, patients receiving both intravenous and nebulized colistin showed faster clinical resolution and higher rates of radiologic improvement than those receiving only intravenous treatment [39].

Combination therapy is increasingly preferred. In CRAB-associated VAP, a retrospective study showed that colistin combined with high-dose tigecycline or carbapenems improved clinical response and reduced ICU mortality compared to colistin alone [42]. In vitro data also support synergistic effects between colistin and antibiotics such as fosfomycin, sulbactam, and rifampin, although evidence from prospective clinical trials remains limited [43].

Resistance to colistin, although still relatively low, is on the rise. A systematic review published in 2024 reported an increase in colistin resistance among *A. baumannii* isolates from 3.2% pre-2015 to 8.9% post-2020, particularly in ICU environments [44]. This underscores the importance of antimicrobial stewardship and combining colistin with other agents when used.

### 5.2. Cefiderocol

Cefiderocol is a novel siderophore cephalosporin that demonstrates potent in vitro activity against multidrug-resistant (MDR) Gram-negative pathogens, including *A. baumannii*. Its mechanism of action relies on active transport through bacterial iron uptake systems and enhanced stability against both serine- and metallo-β-lactamases, including OXA-type carbapenemases [45,46].

Clinical trial data have yielded mixed outcomes regarding cefiderocol’s efficacy against *A. baumannii*. In the randomized controlled APEKS-NP trial, cefiderocol demonstrated non-inferiority to high-dose meropenem in the treatment of nosocomial pneumonia, including infections caused by *A. baumannii* [47]. However, in the open-label CREDIBLE-CR trial, a higher mortality rate was observed in the cefiderocol group compared to best available therapy (BAT), particularly in patients infected with *A. baumannii* [47]. Baseline differences, such as more frequent renal impairment and ICU admission in the cefiderocol group, complicate the interpretation of efficacy results.

Between 2016 and 2019, 152 patients were randomized: 101 received cefiderocol and 51 received the best available therapy. Of these, 150 were treated (101 with cefiderocol, 85% in monotherapy, and 49 with standard therapy, 61% in combination therapy). In the carbapenem-resistant microbiological ITT population (118 patients), the most common pathogens were *Acinetobacter baumannii* (46%), *Klebsiella pneumoniae* (33%), and *Pseudomonas aeruginosa* (19%) [47].

Treatment-emergent adverse events were observed in 91% of the cefiderocol group and 96% of the best available therapy group. Mortality rates were higher in the cefiderocol group (34%) compared to the best available therapy group (18%), with one death directly related to the study drug. Patients with *Acinetobacter* infections treated with cefiderocol had more baseline risk factors, including renal dysfunction and ICU admission [47].

Higher mortality in the cefiderocol group occurred mainly in patients with *Acinetobacter* infections and preexisting risk factors, complicating the interpretation of efficacy data. The small sample size and patient heterogeneity limited the study’s ability to balance baseline factors. Future studies could consider additional stratification factors to better balance mortality risk. The open-label design was necessary due to the variability in best available therapy regimens. The composite endpoint used was confounded by individual components, and further limitations included the inability to adjust for factors affecting outcomes and the mixing of safety and efficacy evaluations [47].

### 5.3. Tigecycline

Tigecycline is a glycylcycline antibiotic with broad-spectrum activity against Gram-negative pathogens, including certain multidrug-resistant *A. baumannii* strains. It inhibits bacterial protein synthesis by binding to the 30S ribosomal subunit. Despite in vitro activity, tigecycline’s role in severe infections is debated due to low serum levels, especially in bloodstream infections [48].

Several studies have investigated the efficacy of high-dose tigecycline (HD-TGC) regimens. In a retrospective cohort of patients with XDR *A. baumannii* bacteremia, Han et al. reported improved outcomes in those treated with high-dose tigecycline (200 mg loading dose followed by 100 mg every 12 h), compared to standard doses [49]. Similarly, a prospective pharmacokinetic study published in 2023 confirmed that high-dose tigecycline achieved better pharmacokinetic targets, though no significant differences were observed in overall mortality or microbiological eradication between dosing groups [50].

A meta-analysis published in 2024 confirmed that high-dose tigecycline [100 mg every 12 h] was significantly more effective than control regimens in VAP due to MDR pathogens, without increasing adverse event rates [51].

Combination therapy involving tigecycline is often pursued to enhance efficacy. A retrospective cohort study found that adding tigecycline to combination therapy improved clinical response and reduced failure in ICU pneumonia cases [52]. In vitro studies also suggest synergistic interactions between tigecycline and polymyxins, especially colistin, against *A. baumannii* [53].

Nonetheless, resistance to tigecycline is increasing, mainly due to the upregulation of efflux pumps [e.g., *AdeABC*, *AdeIJK*] and mutations in ribosomal targets. A narrative review published in 2023 emphasized the need for careful use of tigecycline to preserve its effectiveness in multidrug-resistant infections [54]. Though limited as monotherapy, tigecycline is useful in optimized or combination regimens, especially in salvage cases with few options.

### 5.4. Combination Therapies

Due to the limited efficacy of monotherapy in treating MDR and XDR *A. baumannii* infections, combination regimens are increasingly used in clinical practice. These strategies aim to enhance antibacterial activity, reduce resistance development, and improve outcomes in severe infections such as pneumonia and bacteremia. Although high-quality evidence remains limited, available data support the use of selected combinations, particularly in critically ill patients with few therapeutic options.

#### 5.4.1. Comparative Efficacy: Colistin Plus Tigecycline vs. Colistin Plus Carbapenem

Several clinical and observational studies, as well as meta-analyses, have evaluated outcomes of different colistin-based dual therapies in CRAB infections, though direct head-to-head comparisons remain limited. Overall, the evidence suggests that although combination regimens may improve secondary outcomes compared with monotherapy, there is no consistent evidence that colistin plus tigecycline is superior to colistin plus carbapenem across studies.

A recent retrospective cohort study reported favorable outcomes with both combinations. Clinical cure and microbiological eradication rates were higher than with colistin monotherapy. However, 30-day mortality did not differ significantly between the two combinations [55]. In patients with CRAB pneumonia, another study compared meropenem–colistin and meropenem–tigecycline. The results showed similar mortality rates and adverse event profiles [56]. These findings suggest that both combinations are clinically useful, but no clear superiority was demonstrated.

Pharmacologically, the combinations have distinct advantages. Tigecycline achieves higher concentrations in lung tissue. This makes it suitable for pulmonary infections. In contrast, carbapenems provide better plasma concentrations, favoring their use in bacteremia [57]. Importantly, using colistin in combination can reduce the risk of resistance development. This supports the rationale for avoiding monotherapy in severe CRAB infections [58].

In conclusion, both colistin plus tigecycline and colistin plus carbapenem are reasonable options. The decision should be based on the site of infection, antibiotic susceptibility, and patient-specific factors. Further randomized clinical trials are needed to establish a clear therapeutic advantage.

#### 5.4.2. Ceftazidime/Avibactam

Ceftazidime–avibactam, a combination of a third-generation cephalosporin and a non-β-lactam β-lactamase inhibitor, is approved for the treatment of infections caused by carbapenem-resistant *Enterobacterales* and some *Pseudomonas aeruginosa* strains. However, its activity against *A. baumannii* is significantly limited. Avibactam fails to inhibit OXA-type β-lactamases, the main resistance mechanism in *A. baumannii*, making this combination largely ineffective [59,60].

Resistance to ceftazidime–avibactam in *A. baumannii* is typically intrinsic but can also be mediated by porin mutations and efflux pump overexpression. A systematic analysis reported that more than 50% of *A. baumannii* isolates tested globally between 2015 and 2022 were non-susceptible to ceftazidime–avibactam [61].

Given this limited spectrum and resistance profile, current international guidelines do not recommend ceftazidime–avibactam for the treatment of *A. baumannii* infections. Its use remains investigational in this context and may only be considered in exceptional cases involving co-infections or as part of salvage regimens guided by susceptibility results [62].

#### 5.4.3. Sulbactam–Durlobactam

Sulbactam–durlobactam (SUL–DUR) is a newly approved combination consisting of sulbactam, a β-lactam antibiotic with intrinsic activity against *Acinetobacter* spp., and durlobactam, a novel diazabicyclooctane β-lactamase inhibitor. Sulbactam exerts its activity by binding to penicillin-binding proteins [PBPs], particularly PBP1a, PBP1b, and PBP3, while durlobactam inhibits class A, C, and D β-lactamases, including the clinically significant OXA-type enzymes that mediate carbapenem resistance in *A. baumannii* [63].

The combination has shown promising in vitro activity against a broad range of multidrug-resistant *A. baumannii* isolates, including those resistant to carbapenems, colistin, and aminoglycosides [64]. The pivotal ATTACK trial, a randomized, controlled, phase 3 study, demonstrated non-inferiority of SUL–DUR compared to colistin in patients with serious infections caused by *A. baumannii-calcoaceticus* complex. Notably, SUL–DUR was associated with a significantly lower rate of nephrotoxicity (13.2% vs. 37.6%) while achieving similar clinical cure and 28-day mortality rates [65].

These findings position SUL-DUR as a valuable therapeutic option for infections due to CRAB, particularly in critically ill patients at risk for renal injury. However, real-world effectiveness data remain limited, and ongoing surveillance is needed to monitor for emerging resistance under therapeutic pressure.

Given the increasing complexity of resistance mechanisms and the growing number of therapeutic options under evaluation, a structured comparison of key agents may assist clinicians in selecting appropriate regimens. Table 6 summarizes the main pharmacological and clinical features of both established and emerging treatments for *A. baumannii* infections.

## 6. Prevention

Effective prevention of *A. baumannii* pneumonia in hospital settings hinges on a multimodal infection prevention and control (IPC) bundle.

Hand hygiene remains essential, with improved compliance among healthcare workers associated with a notable annual reduction in CRAB incidence (≈9.8%), as shown in cohort studies based on direct observation [56].

Active screening and surveillance cultures in high-risk areas such as ICUs allow early detection of colonized patients, enabling prompt isolation or cohorting measures [66].

Environmental cleaning and surface disinfection, including regular rotation of disinfectants during outbreaks, has proven effective in controlling CRAB transmission in ICUs [67,68].

Contact precautions and isolation, such as single-room placement or cohorting CRAB-positive patients, are recommended in international guidelines to prevent respiratory cross-transmission [66].

Finally, antimicrobial stewardship is critical. Approaches like de-escalation based on culture results, shorter durations of broad-spectrum therapy, and frequent clinical reassessment have been shown to be safe in VAP and help reduce resistance pressure [69].

## 7. Conclusions

In conclusion, the management of *A. baumannii* infections—particularly those caused by MDR and XDR strains—remains a major challenge due to limited therapeutic options and rising resistance. While novel antimicrobials and optimized combination regimens offer new hope, their success is inherently linked to rapid diagnostics, antimicrobial stewardship, and timely clinical decision-making. Crucially, preventive strategies such as infection control, surveillance, and hygiene measures are essential pillars in curbing the spread of MDR-AB. Without coordinated action that couples effective infection prevention with stewardship, rapid diagnostics, and access to active therapies, MDR/XDR *A. baumannii* will continue to drive avoidable morbidity and mortality in high-risk hospital settings.

## Figures and Tables

**Table 1:** Summary of key Romanian studies on antimicrobial resistance in *A. baumannii* (2011–2025) [25,26,27,28].

Region/Context	No. of Isolates	Methodology/Testing	Main Findings
Multicenter (Romania, 2025)	142 clinical isolates from 6 hospitals	Phenotypic AST (EUCAST); genotyping by PCR and WGS	Approximately 91.5% of *A. baumannii* isolates were extensively drug-resistant and carbapenem-resistant (XDR-CRAB). The predominant genes were bla_OXA-23-like (91.5%), bla_OXA-24/40-like (74.6%), and ArmA (63.6%) [25].
Bucharest (Tertiary Infectious Disease Hospital, 2017–2024)	289 bloodstream isolates	Automated susceptibility testing (VITEK 2)	Bloodstream isolates of *A. baumannii* showed 100% resistance to carbapenems and aminoglycosides by 2024; overall MDR prevalence ≈ 90.7% [26].
Western Romania (ICU, Timișoara, 2011–2015)	185 ICU isolates	Disk diffusion (CLSI); confirmatory E-test	*A. baumannii* isolates exhibited 94.6% resistance to both imipenem and ceftazidime; 81.1% to ampicillin/sulbactam [27].
Clinical and Environmental Isolates (2018–2019)	70 clinical + 28 wastewater isolates	Phenotypic AST + PCR for resistance genes	Both hospital and wastewater *A. baumannii* isolates carried multiple resistance genes (bla_OXA-23, bla_OXA-24, bla_SHV, bla_TEM, bla_GES), suggesting cross-contamination between hospitals and environment [28].

AST—Antimicrobial susceptibility testing; CRAB—Carbapenem-resistant *Acinetobacter baumannii*; XDR—Extensively drug-resistant, EUCAST—European Committee on Antimicrobial Susceptibility Testing; PCR—Polymerase Chain Reaction; WGS—Whole genome sequencing; VITEK 2—automated identification and antimicrobial susceptibility testing system; MDR—Multidrug-Resistant; ICU—Intensive Care Unit; CLSI—Clinical and Laboratory Standards Institute; OXA—Oxacillinase; ArmA—Aminoglycoside resistance methyltransferase; bla_SHV—gene encoding SHV (sulfhydryl reagent variable) β-lactamase; bla_TEM—gene encoding TEM (Temoniera) β-lactamase; bla_GES—gene encoding GES (Guiana extended-spectrum) β-lactamase.

**Table 2:** Overview of resistance mechanisms and associated antibiotics in *Acinetobacter baumannii*. Data adapted and synthesized from Černiauskienė et al. [24], under CC BY 4.0 license.

Class	Enzyme Type	Mechanism	Example Enzymes	Inhibition by β-Lactamase Inhibitors?	Found in *A. baumannii*
A	Serine β-lactamase	Hydrolyzes via serine site	KPC, TEM, SHV	Yes	Occasional (ESBL TEM/SHV); KPC very rare
B	Metallo-β-lactamase	Zinc-dependent hydrolysis	NDM, VIM, IMP	Limited/variable	Common (intrinsic ADC)
C	AmpC cephalosporinase	Chromosomal or plasmidic cephalosporinase	CMY, FOX	Limited/variable	Rare
D	Oxacillinases (OXA)	Hydrolyzes oxacillin and carbapenems	OXA-23, OXA-24/40, OXA-58, OXA-51 like	Limited/variable	Common

KPC—*Klebsiella pneumoniae* Carbapenemase; TEM—Temoneira β-lactamase; SHV—Sulfhydryl variable; ESBL—Extended-Spectrum Beta-Lactamase; NDM—New Delhi metallo-β-lactamase; VIM—Verona integron-encoded metallo-β-lactamase; IMP—Imipenemase; ADC—*Acinetobacter*-Derived Cephalosporinase, AmpC—Class C cephalosporinase, CMY—Cephamycin; FOX—Forkhead box protein; OXA—Oxacillinase.

**Table 3:** Clinically relevant carbapenemases detected in *Acinetobacter baumannii* strains. Adapted from Mancuso et al. [29] and Černiauskienė et al. [32].

Carbapenemase [Gene]	Carbapenem Activity	Prevalence	Notes
OXA-23	High	Global, high in Asia and Europe	Most prevalent; often associated with ISAba1 insertions
OXA-24/40	Moderate to High	Europe, sporadic elsewhere	Plasmid or chromosomal; potent but less widespread
OXA-58	Variable	Outbreak-related	Frequently plasmid-mediated; involved in nosocomial outbreaks
OXA-51-like	Low unless upregulated	Intrinsic to all *A. baumannii*	Chromosomal; expression level determines resistance
NDM-1 [29]	Very High	Emerging globally, rare in *A. baumannii*	Requires zinc; not inhibited by classical inhibitors

OXA—Oxacillinase; NDM—New Delhi metallo-β-lactamase; ISAba1—Insertion Sequence Aba1.

**Table 4:** Major efflux pump systems and associated resistance profiles in *Acinetobacter baumannii*. Efflux data summarized based on Choquet et al. [34] and Xie et al. [35].

Efflux Pump System	Superfamily	Genes Involved	Antibiotics Affected
AdeABC [34,35]	RND	*adeA*, *adeB*, *adeC*	Aminoglycosides, β-lactams, fluoroquinolones, tigecycline
AdeIJK [34,35]	RND	*adeI*, *adeJ*, *adeK*	Chloramphenicol, tetracycline, fluoroquinolones
AdeFGH [34,35]	RND	*adeF*, *adeG*, *adeH*	Chloramphenicol, tigecycline, trimethoprim
CraA [34]	MFS	*craA*	Chloramphenicol
AmvA [34]	MFS	*amvA*	Disinfectants, dyes
AbeM [34]	MATE	*abeM*	Fluoroquinolones, gentamicin

AdeABC/AdeIJK/AdeFGH- Multidrug efflux pump system; CraA—Chloramphenicol Resistance *Acinetobacter*; AmvA—*Acinetobacter* multiple valence efflux type A; *Acinetobacter* efflux pump type MATE; RND—Resistance Nodulation Division; MFS—Major Facilitator Superfamily; AbeM—*Acinetobacter* efflux pump type MATE; MATE—Multidrug and Toxic Compound Extrusion.

**Table 5:** Outer membrane porins in *Acinetobacter baumannii* and their roles in antibiotic resistance. Adapted from Sarshar et al. [36] and Rangel et al. [9], based on structural and functional analyses of outer membrane proteins.

Porin	Function	Resistance Mechanism	Antibiotics Affected
OmpA [36]	Structural OMP; adhesion and biofilm	Minor role in permeability and biofilm-related tolerance	Multiple, including β-lactams
Omp33–36 [9]	Non-specific diffusion channel	Downregulation reduces β-lactam and carbapenem influx	Carbapenems, cephalosporins
CarO [36]	Facilitates imipenem uptake	Gene disruption or mutation leads to decreased permeability	Primarily imipenem
OprD [9]	Basic amino acids/antibiotic uptake	Often altered or downregulated in resistant strains	Carbapenems (primarily described in *Pseudomonas aeruginosa*; evidence in *A. baumannii* is limited)

OmpA—Outer Membrane Protein A; Omp33-36—Outer membrane porin 33–36 kDa; CarO—Carbapenem-Associated outer membrane protein O; OprD—Outer membrane porin D.

**Table 6:** Summary of antimicrobial agents used in the treatment of *Acinetobacter baumannii* infections, including pharmacological features, MIC ranges, and adverse events.

Antibiotic/Combination	Mechanism of Action	MIC Range [µg/mL]	Level of Evidence	Adverse Effects
Colistin (Polymyxin E)	Disrupts bacterial membranes	0.5–2 (susceptible)	Moderate (observational, in vitro)	Nephrotoxicity, neurotoxicity
Tigecycline	Inhibits protein synthesis (30S ribosome)	0.25–2 (variable)	Low (retrospective studies)	Nausea, vomiting, hepatotoxicity
Carbapenems (e.g., meropenem)	Inhibits cell wall synthesis	>8 (often resistant)	Moderate (clinical practice, guidelines)	Seizures (especially imipenem), nephrotoxicity
Cefiderocol	Siderophore cephalosporin	0.12–4	High (clinical trials)	Diarrhea, infusion site reactions
Sulbactam–Durlobactam	β-lactamase inhibitor + β-lactam	1–4	High (Phase 3 trials)	Mild gastrointestinal disturbances
Ampicillin–Sulbactam	Cell wall inhibition + β-lactamase block	2–8	Moderate (clinical use)	Hepatotoxicity, rash
Ceftazidime–Avibactam	Cephalosporin + β-lactamase inhibitor	>16	Low (limited studies for *A. baumannii*)	Hypersensitivity, diarrhea
Polymyxin B	Similar to colistin	0.25–2	Moderate (clinical use, observational)	Nephrotoxicity, neurotoxicity

MIC—minimum inhibitory concentration; Level of Evidence: based on available trial data or guidelines.

## Data Availability

The data published in this research are available on request from the first author and corresponding author. All information provided in this review is documented by relevant references.

## Abbreviations

AB	*Acinetobacter baumannii*
AbeM	*Acinetobacter* efflux pump type MATE
ACB	*Acinetobacter calcoaceticus–baumannii*
ADC	*Acinetobacter*-Derived Cephalosporinase
AdeABC	Multidrug efflux pump system
AmpC	Class C cephalosporinase
AMR	Antimicrobial Resistance
AmvA	*Acinetobacter* multiple valence efflux type A
APEKS-NP	*Acinetobacter-Pseudomonas* Evaluation for Carbapenem Susceptibility—Non-fermenter Panel
ArmA	Aminoglycoside resistance methyltransferase
APC	Article Processing Charge
AST	Antimicrobial Susceptibility Testing
ATTACK	*Acinetobacter* Treatment Trial Against Colistin-Resistant A. *baumannii*
blaSHV	Beta-lactamase sulfhydryl reagent variable
blaGES	Beta-lactamase Guiana extended-spectrum
BL/BLI	Beta-Lactam/Beta-Lactamase Inhibitor
CAP	Community-acquired pneumonia
CarO	Carbapenem-Associated outer membrane protein O
CFDC	Cefiderocol
CLSI	Clinical and Laboratory Standards Institute
CMY	Cephamycin
CraA	Chloramphenicol Resistance *Acinetobacter*
CRAB	Carbapenem-Resistant *Acinetobacter baumannii*
CREDIBLE-CR	Ceftazidime–Avibactam in the Treatment of Serious Infections due to Carbapenem-Resistant Gram-Negative Pathogens
DNA	Deoxyribonucleic Acid
ESBL	Extended-Spectrum Beta-Lactamase
EUCAST	European Committee on Antimicrobial Susceptibility Testing
FOX	Forkhead box protein
gyrA	DNA gyrase subunit A
gyrB	DNA gyrase subunit B
HAP	Hospital-Acquired Pneumonia
HD-TGC	High-dose Tigecycline
ICU	Intensive Care Unit
IMP	Imipenemase
IS	Insertion Sequence
ISAba1	Insertion Sequence Aba1
KPC	*Klebsiella pneumoniae* Carbapenemase
LPS	Lipopolysaccharide
MATE	Multidrug and Toxic Compound Extrusion
MBLs	Metallo-β-lactamases
MDR	Multidrug-Resistant
MFS	Major Facilitator Superfamily
MIC	Minimum Inhibitory Concentration
MLST	Multilocus Sequence Typing
NDM	New Delhi metallo-β-lactamase
OmpA	Outer Membrane Protein A
Omp33-36	Outer membrane porin 33–36 kDa
OprD	Outer membrane porin D
OXA	Oxacillinase
parC	Topoisomerase IV subunit A gene
parE	Topoisomerase IV subunit B gene
PBPs	Penicillin-binding proteins
PCR	Polymerase Chain Reaction
PDR	Pandrug resistance
PK/PD	Pharmacokinetics / Pharmacodynamics
QRDRs	Quinolone resistance-determining regions
RND	Resistance Nodulation Division
SHV	Sulfhydryl variable
SUL–DUR	Sulbactam–durlobactam
TEM	Temoneira β-lactamase
VAP	Ventilator-associated pneumonia
VIM	Verona integron-encoded metallo-β-lactamase
WHO	World Health Organisation
XDR	Extensively drug-resistant
WGS	Whole Genome Sequencing
VITEK 2	Automated identification and antimicrobial susceptibility testing system
